# Supplementary figures and images for: Proteome remodeling in the zoospore-to-vegetative cell transition of the stramenopile Aurantiochytrium limacinum reveals candidate ectoplasmic network proteins
Source: PLoS One. 2025 Jul 2;20(7):e0326651. doi: 10.1371/journal.pone.0326651 (PMC12221091; doi:10.1371/journal.pone.0326651)

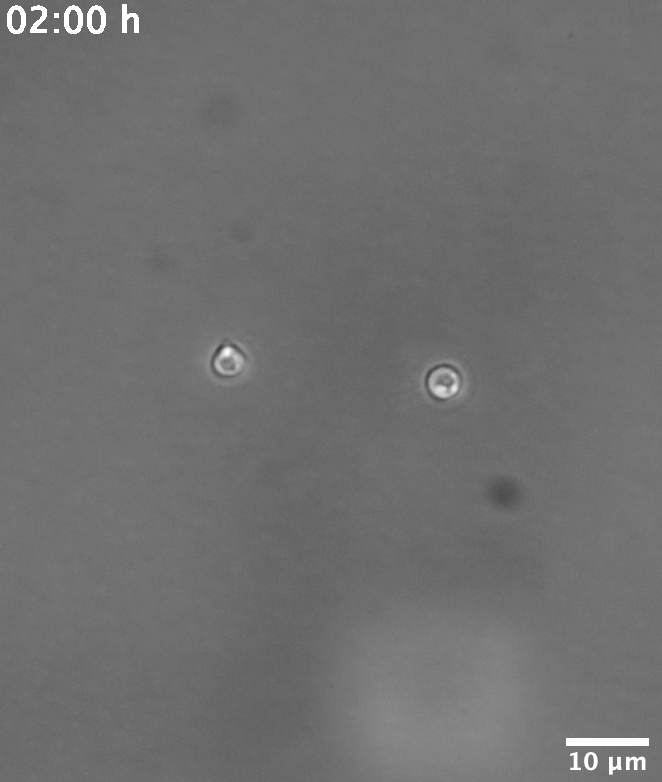

Supplement: S1 File — Compressed in zip format. (ZIP) [file pone.0326651.s001.zip › t2.tif]

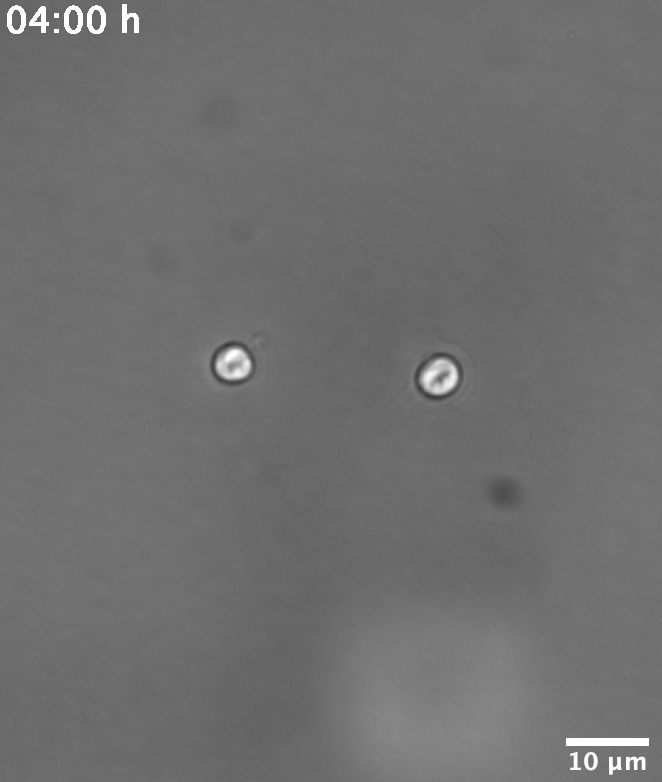

Supplement: S1 File — Compressed in zip format. (ZIP) [file pone.0326651.s001.zip › t4.tif]

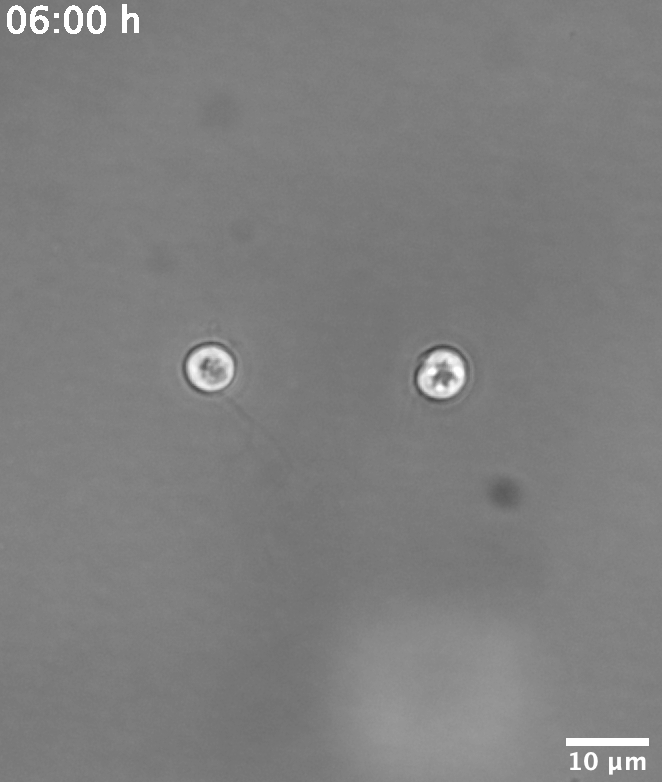

Supplement: S1 File — Compressed in zip format. (ZIP) [file pone.0326651.s001.zip › t6.tif]

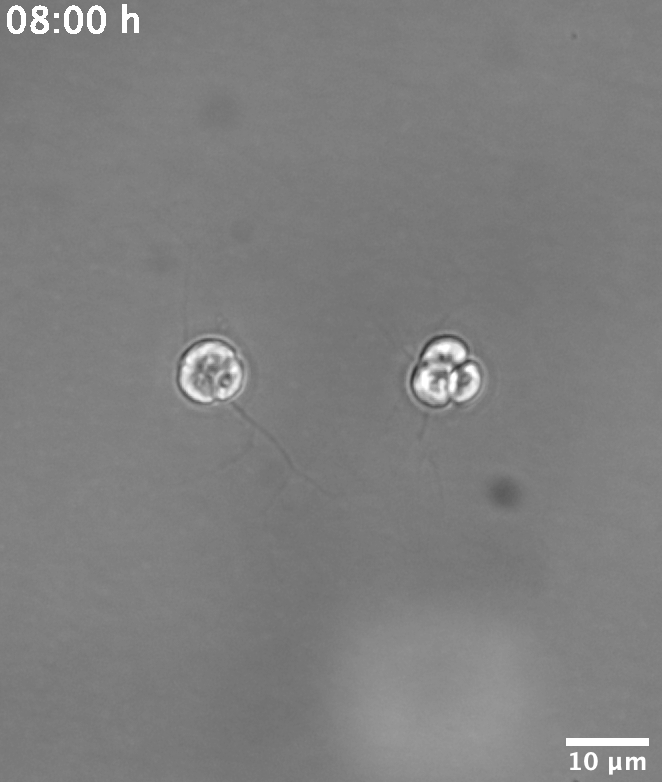

Supplement: S1 File — Compressed in zip format. (ZIP) [file pone.0326651.s001.zip › t8.tif]

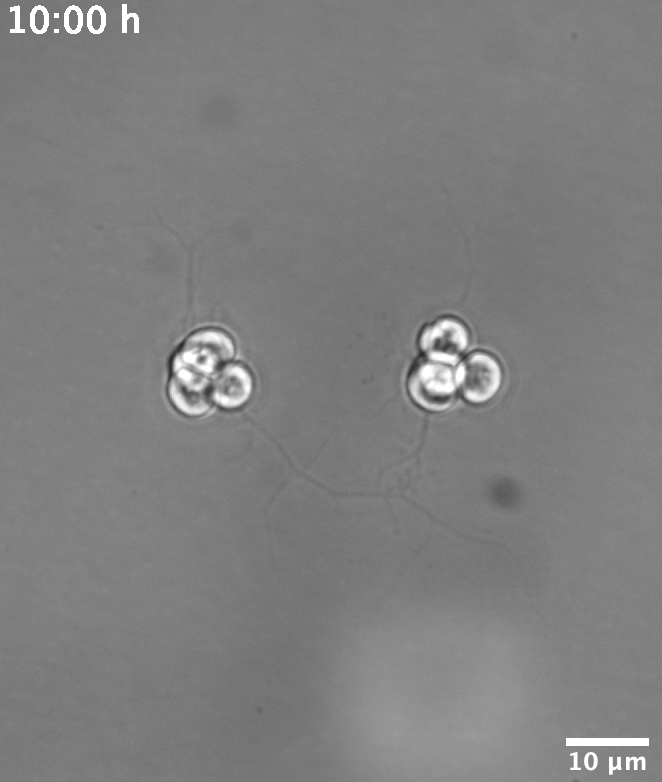

Supplement: S1 File — Compressed in zip format. (ZIP) [file pone.0326651.s001.zip › t10.tif]

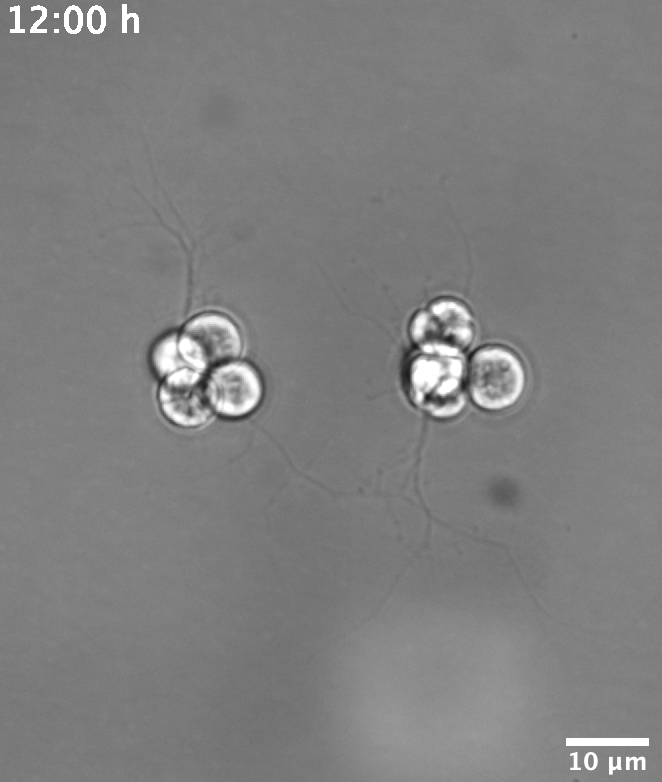

Supplement: S1 File — Compressed in zip format. (ZIP) [file pone.0326651.s001.zip › t12.tif]
