# Supplementary material for: Proteome remodeling in the zoospore-to-vegetative cell transition of the stramenopile Aurantiochytrium limacinum reveals candidate ectoplasmic network proteins
Source: PLoS One. 2025 Jul 2;20(7):e0326651. doi: 10.1371/journal.pone.0326651 (PMC12221091; doi:10.1371/journal.pone.0326651)

Supplemental Figures for

# Proteome remodeling in the zoospore-to-vegetative cell transition of the stramenopile *Aurantiochytrium limacinum* reveals candidate ectoplasmic network proteins

Alejandro Gil-Gomez, Ben Leyland, Anbarasu Karthikaichamy, Rebecca C. Adikes, David Q. Matus, Joshua S. Rest, Jackie L. Collier

Legends for supporting files and tables
- S1 File
- S1 Table legends
- S2 Table
- S3 Table
- S4 Table

S1 Figure
S2 Figure
S3 Figure
S4 Figure
S5 Figure
S6 Figure
S7 Figure
S8 Figure
- S8A Figure
- S8B Figure
- S8C Figure
- S8D Figure
- S8E Figure
- S8F Figure
- S8G Figure
- S8H Figure
- S8I Figure
- S8J Figure

# Legends for supporting files and tables

## S1 File

**S1 File. Original stills from time-lapse video microscopy used to make Fig 1.** Compressed in zip format.

## S1 Table legends

**S1A Table. Time course of zoospore settlement and vegetative cell development.** Observations by inverted microscopy from a representative experiment of freshly isolated *A. limacinum* zoospores (200 microliters) inoculated into 10 ml A1 media in T25 flask; temperature was 74.2 F.

**S1B Table. The 20 most abundant proteins in the detected dataset across all time points and replicates.** Shown are protein ID and cluster membership where applicable, maximum log2 expression value, and KEGG annotation.

**S1C Table. Differential representation (Fisher Exact Test) analysis for KOG classes and ko groups.** ‘det’ is the number of detected proteins annotated in each ko group or KOG class; ‘sig’ is the number of detected proteins that were significantly different from T0 at any timepoint in the merged dataset; ‘C1’, ‘C2’, ‘C3’, ‘C4’ are the number of significant proteins in each cluster in the heatmap Figure 5; ‘down’ and ‘up’ are the sum of proteins in C1 and C2 or C3 and C4, respectively. The columns ‘p’ give the Benjamini-Hochberg-corrected (false discovery rate 5 %) p-values for the previous 6 columns from Fisher’s Exact Test, and columns ‘t’ summarize the direction of significant results. *Categories excluded from Fig. 6 because they are hard to interpret.

**S1D Table. GO terms significantly ($p<0.01$) overrepresented in each cluster or downregulated (C1 plus C2) or upregulated (C3 plus C4) in weighted GO analysis.** ‘Annotated’, ‘Significant’, and ‘Expected’ are numbers of proteins; ‘type’ refers to GO ontology, ‘annotation1’, ‘annotation 2’, and ‘otherNotes’ are categorizations used to organize and interpret the data.

**S1E Table. Details of bioinformatic analysis of candidate EN/bothrosome proteins.**

**S1F Table. Summary of proteins not represented in Venn diagrams (S2 and S3 Figures) due to complex mapping between JGI and MMETSP datasets.**

**S1G Table. Comparison of significantly differential proteins identified in this study with significantly differential transcripts identified by Dellero et al. 2020.**
In each column, 'C1 or C2' indicates proteins enriched in zoospores while 'C3 or C4' indicates proteins enriched in vegetative cells, and 'mRNA up' indicates transcripts enriched in zoospores while 'mRNA down' indicates transcripts enriched in vegetative cells based on Dellero et al.

(2020). In each row, 'yes' indicates which category an individual protein is found in, 'ns' indicates a non-significant protein mentioned in the text but significant in Dellero, and integers indicate how many genes belonging to ko or KOG groups are in each category. Correspondence between our proteomic results and Dellero's RNA-seq results are provided in columns KL to KS in **S2 Table**.

## S2 Table

**S2 Table. Proteomic differential expression analysis and annotation spreadsheet.** Please see the 'ReadMe' page of the excel file for explanation of each column in the main data page.

## S3 Table

**S3 Table. Proteomics data analyzed against *Aurantiochytrium limacinum* MMETSP predicted proteome.** Available at Dryad DOI https://doi.org/10.5061/dryad.2z34tmpxj

## S4 Table

**S4 Table. Proteomics data analyzed against *Aurantiochytrium limacinum* JGI Aurli1 predicted proteome.** Available at Dryad DOI https://doi.org/10.5061/dryad.2z34tmpxj

# S1 Figure

**S1 Figure. Distribution of intensity and log2-fold changes for all 3783 proteins detected**.

A. Distribution of log2 intensity values per replicate and time point.

B. Distribution of the log2 fold change ratios per time point relative to time point 0.

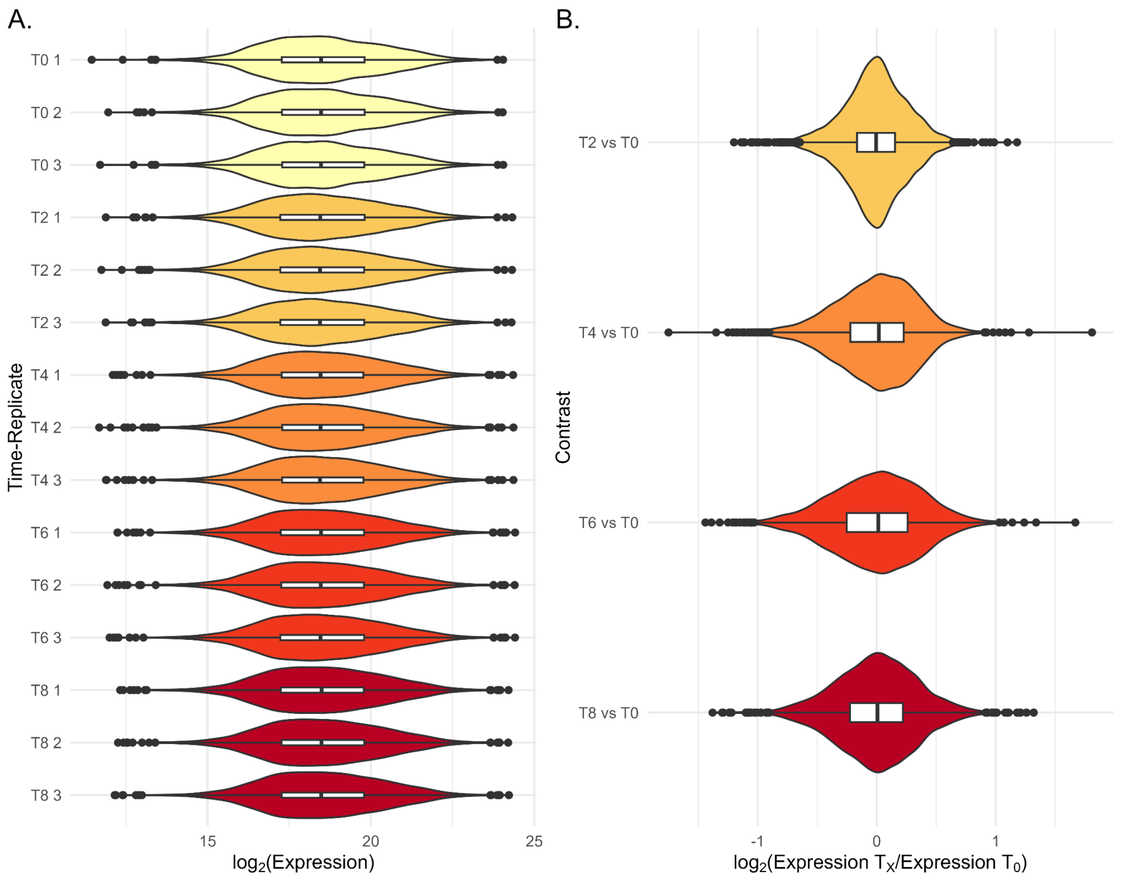

# S2 Figure

**S2 Figure. Overlap between the detected and significant proteins in the JGI vs MMETSP predicted proteomes of *Aurantiochytrium limacinum*.** These figures represent a simplification of complex relationships caused because multiple MMETSP proteins sometimes map to the same JGI; see S4 Figure and S1F Table for details.
A. Overlap between the JGI and MMETSP detected protein sets.
B. Overlap between the JGI and MMETSP significant protein sets.

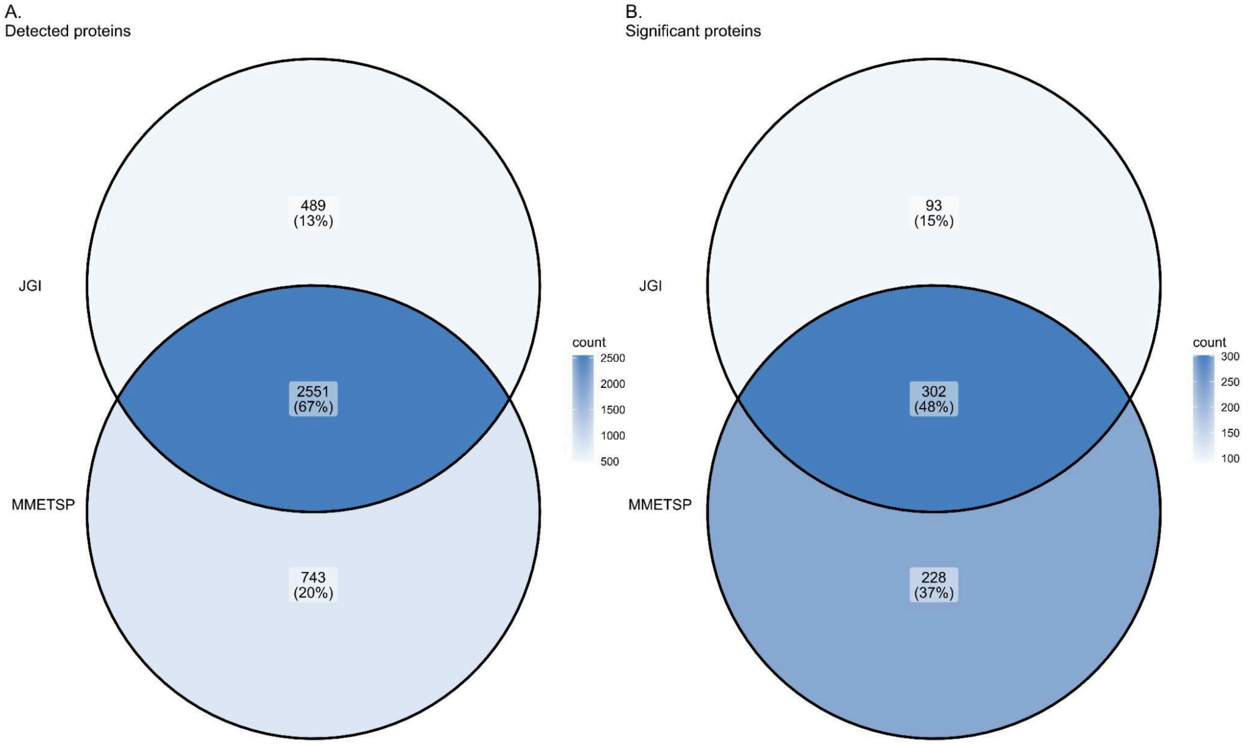

S3 Figure

**S3 Figure. Patterns in significant proteins across time points.** These figures represent a simplification of complex relationships caused because multiple MMETSP proteins sometimes map to the same JGI; see S4 Figure and S1F Table for details.

A. Distribution of 623 differentially expressed proteins across different time points.

B. Distribution of 356 proteins that increased in relative abundance compared to zoospores (T0) across different time points.

C. Distribution of 267 proteins that decreased in relative abundance compared to zoospores (T0) across time points.

A.

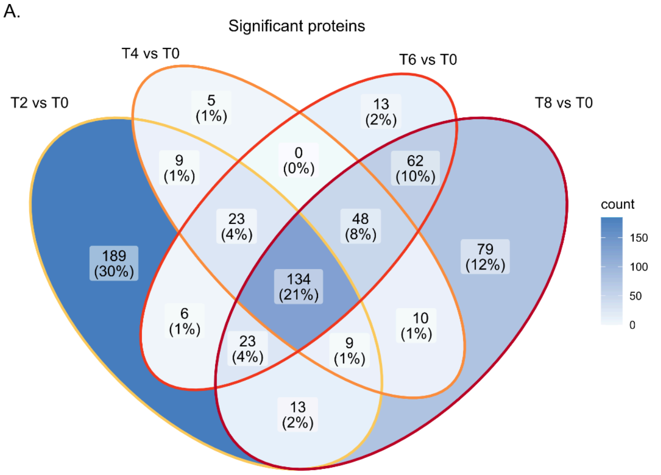


B.

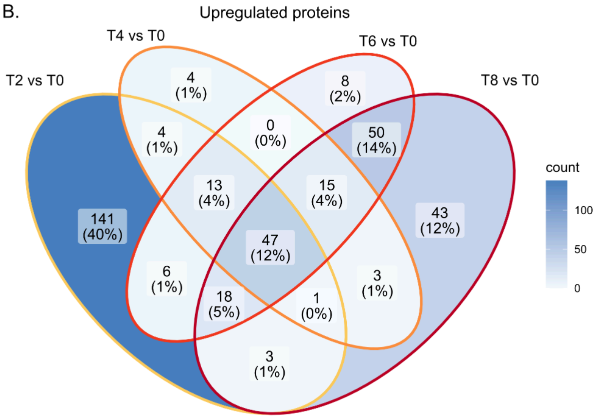


C.

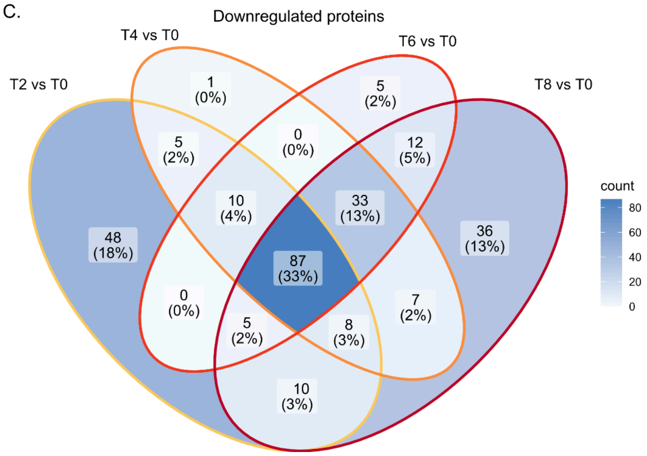

## S4 Figure

**S4 Figure. Comparison of proteins identified in the JGI and MMETSP predicted proteomes.** Each MMETSP protein is mapped to a single predicted JGI protein, but a single JGI protein can be associated with multiple MMETSP proteins. This one-to-many relationship complicates direct comparisons between sets. Each column represents the total number of proteins in different categories. Single-mapped proteins (groups A–J) can be directly counted in their respective categories. However, multi-mapped proteins (groups K–P) may have different totals depending on the annotation method used, due to the complexity introduced by one-to-many mapping. Rows indicate the total number of proteins in each group based on detection and significance across sets: M and J (Detected in both MMETSP and JGI sets), M or J: (Detected in either MMETSP or JGI). SS, S0, 0S, 00: Indicate significance patterns—SS (significant in both M and J), S0 (significant only in M), 0S (significant only in J), and 00 (significant in neither). The final detected set was generated by combining proteins unique to one set with those found in both. For proteins detected in both sets, the version from the set where it was significant was chosen. If a protein was significant in both sets or neither, the version from the MMETSP set was chosen. See S1F Table for more detail on the MMETSP that map to the same JGI.

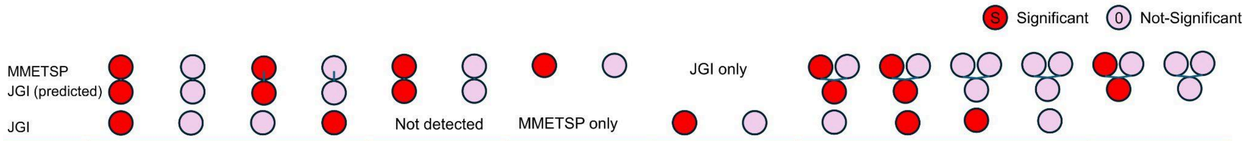


| Category | Single-mapped Detected in JGI | | | | Single-mapped Not detected in J | | No-match | | Only detected in JGI | | Multi-mapped Detected in JGI | | | | Multi-mapped not detected | |
|---|---|---|---|---|---|---|---|---|---|---|---|---|---|---|---|---|
| Group | A | B | C | D | E | F | G | H | I | J | K | L | M | N | O | P |
| M 3298, 530 | 295 | 1990 | 148 | 50 | 31 | 392 | 43 | 266 | NA | NA | 4; 3 | 12; 7 | 6 | 50 | 7; 3 | 4 |
| J(pred) | 295 | 1990 | 148 | 50 | 31 | 392 | NA | NA | NA | NA | 2 | 6 | 2 | 24 | 3 | 2 |
| J 3006, 394 | 295 | 1990 | 148 | 50 | NA | NA | NA | NA | 41 | 448 | 2 | 6 | 2 | 24 | NA | NA |
| **Cat.Totals** | **2483** | | | | **423** | | **309** | | **489** | | **68*** | | | | **11** | |
| M&J SS: 307 | 295 | | | | | | | | | | | 12 | | | | |
| M&J S0: 152 | | | 148 | | | | | | | | 4 | | | | | |
| M&J 0S: 52 | | | | 50 | | | | | | | | | 2 | | | |
| M&J 00: 2040 | | 1990 | | | | | | | | | | | | 50 | | |
| **M&J ALL: 2551** | **295** | **1990** | **148** | **50** | | | | | | | **4** | **12** | **2** | **50** | | |
| M S: 81 | | | | | 31 | | 43 | | | | | | | | 7 | |
| M 0: 662 | | | | | | 392 | | 266 | | | | | | | | 4 |
| **M ALL: 743** | | | | | **31** | **392** | **43** | **266** | | | | | | | **7** | **4** |
| J S: 41 | | | | | | | | | 41 | | | | | | | |
| J 0: 448 | | | | | | | | | | 448 | | | | | | |
| **J ALL: 489** | | | | | | | | | **41** | **448** | | | | | | |
| Total Sign: 623 | 295 | | 148 | 50 | 31 | | 43 | | 41 | | 4 | 12 | 2 | | 7 | |
| **Total Det: 3783** | **295** | **1990** | **148** | **50** | **31** | **392** | **43** | **266** | **41** | **448** | **4** | **12** | **2** | **50** | **7** | **4** |

## S5 Figure

**S5 Figure. Volcano plots colored by heatmap clusters.** The dotted horizontal line represents the significance threshold for each of the time points; everything above this line is significant for that time point. Proteins that are gray are not significant in any of the time points. Proteins that are colored are significant for at least one of the time points.

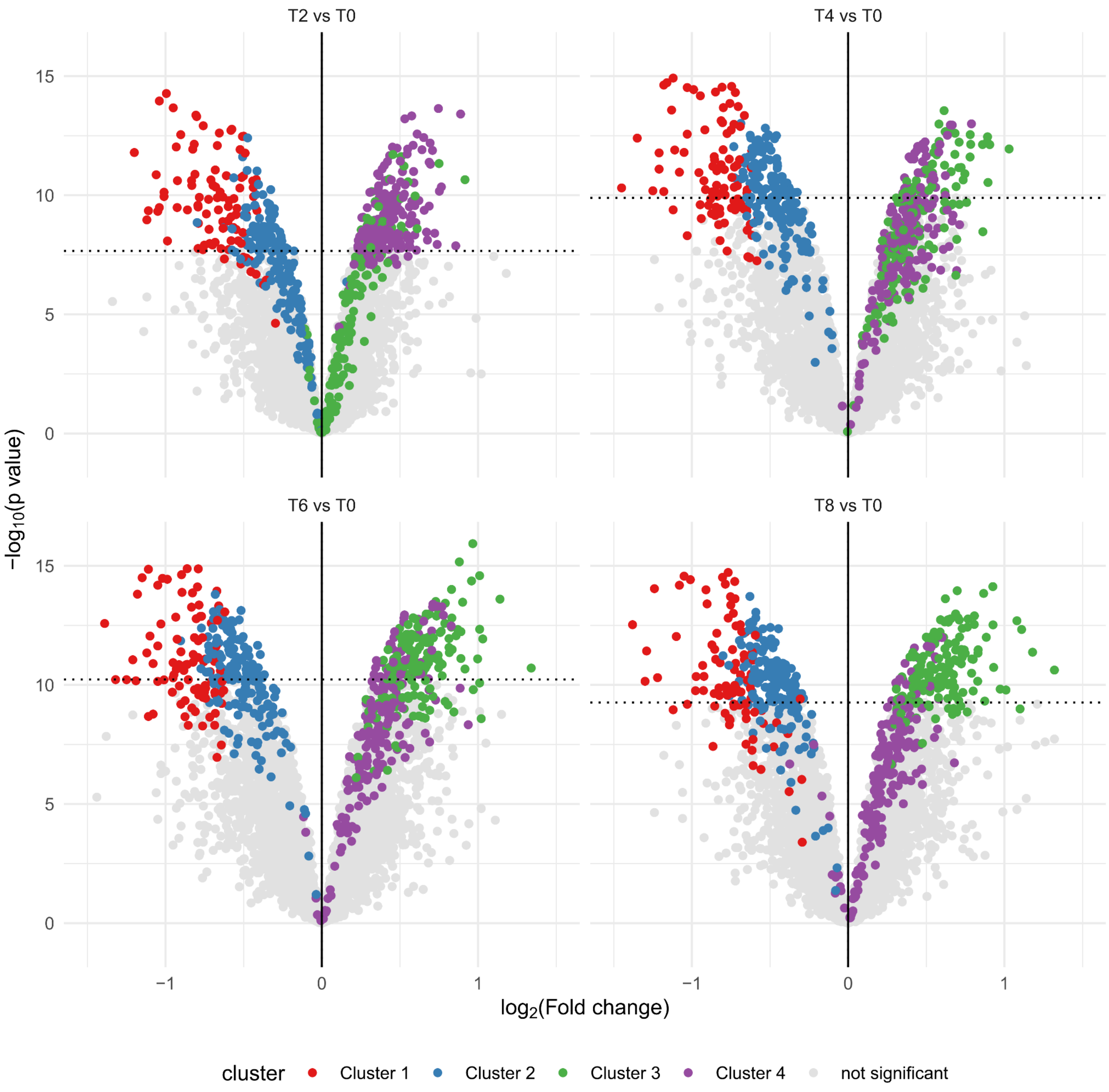

# S6 Figure

**S6 Figure. Full enrichment analysis of KOG and ko functional groups among differentially expressed proteins during the transition from zoospores to vegetative cells in *A. limacinum*..** Bars show the total number of significantly upregulated (red, right-facing) and downregulated (blue, left-facing) proteins (across all time points) relative to zoospores (time 0) for each KOG class or ko group with a significant difference. Symbols at right indicate significant overrepresentation (filled upward triangle) or underrepresentation (open downward triangle) of each class/group among all differentially expressed proteins (test_sig_vs_det), or differentially expressed proteins in clusters C1, C2, C3, and C4 (Fisher's Exact Test with 5 % false discovery rate; columns test_clu1_vs_sig, test_clu2_vs_sig, test_clu3_vs_sig, test_clu4_vs_sig, respectively). Symbols in the last column (test_up_vs_sig) indicate overrepresentation of a class/group among upregulated (C3 and C4, filled upward triangle) or downregulated (C1 and C2, open downward triangle) genes in vegetative cells in comparison to zoospores (Fisher's Exact Test with 5 % false discovery rate).

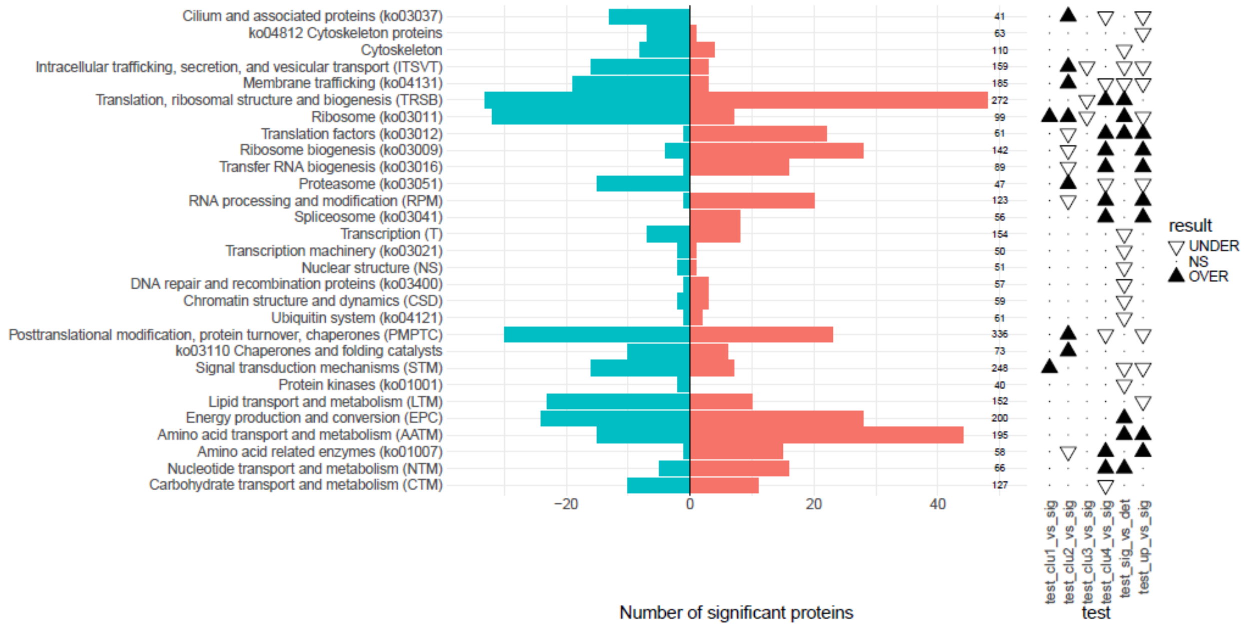

# S7 Figure

**S7 Figure. Volcano plots for KOG class 'translation, ribosomal structure, and biogenesis' colored by ko group.** The line represents the significance threshold for each of the time points, everything above this line is significant for that time point. Proteins are colored by ko: ko03009 Ribosome biogenesis, ko03011 Ribosome, ko03012 Translation factors, ko03016 Transfer RNA biogenesis. Gray proteins belong to other kos.

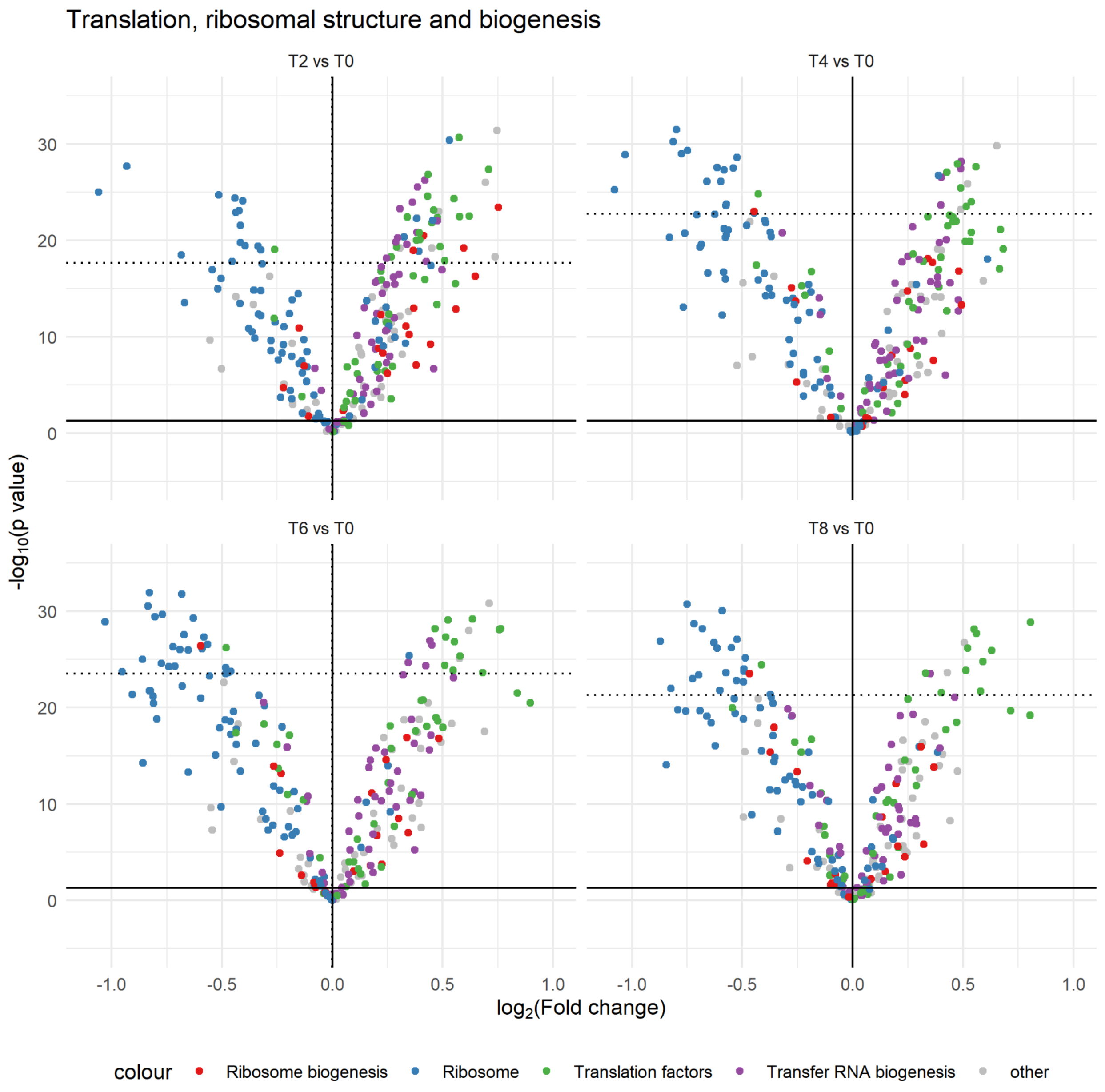

# S8 Figure

## S8A Figure

**S8A Figure.  KEGG map03010, ribosome.** Proteins detected indicated as purple, proteins significantly downregulated at any timepoint indicated as blue, proteins significantly upregulated at any timepoint indicated as red.

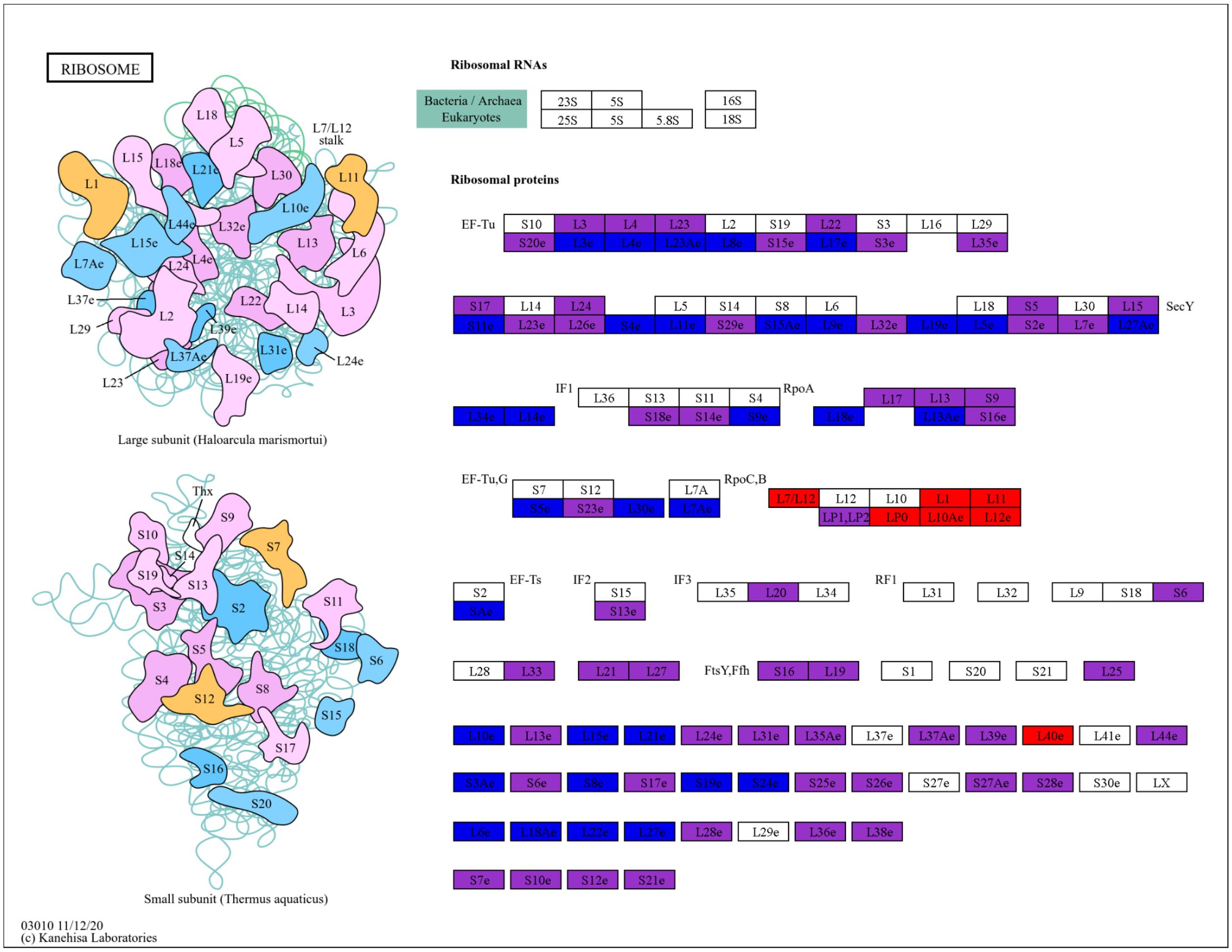

S8B Figure

**S8B Figure. KEGG map03050, proteasome.** Proteins detected indicated as purple, proteins significantly downregulated at any timepoint indicated as blue, proteins significantly upregulated at any timepoint indicated as red.

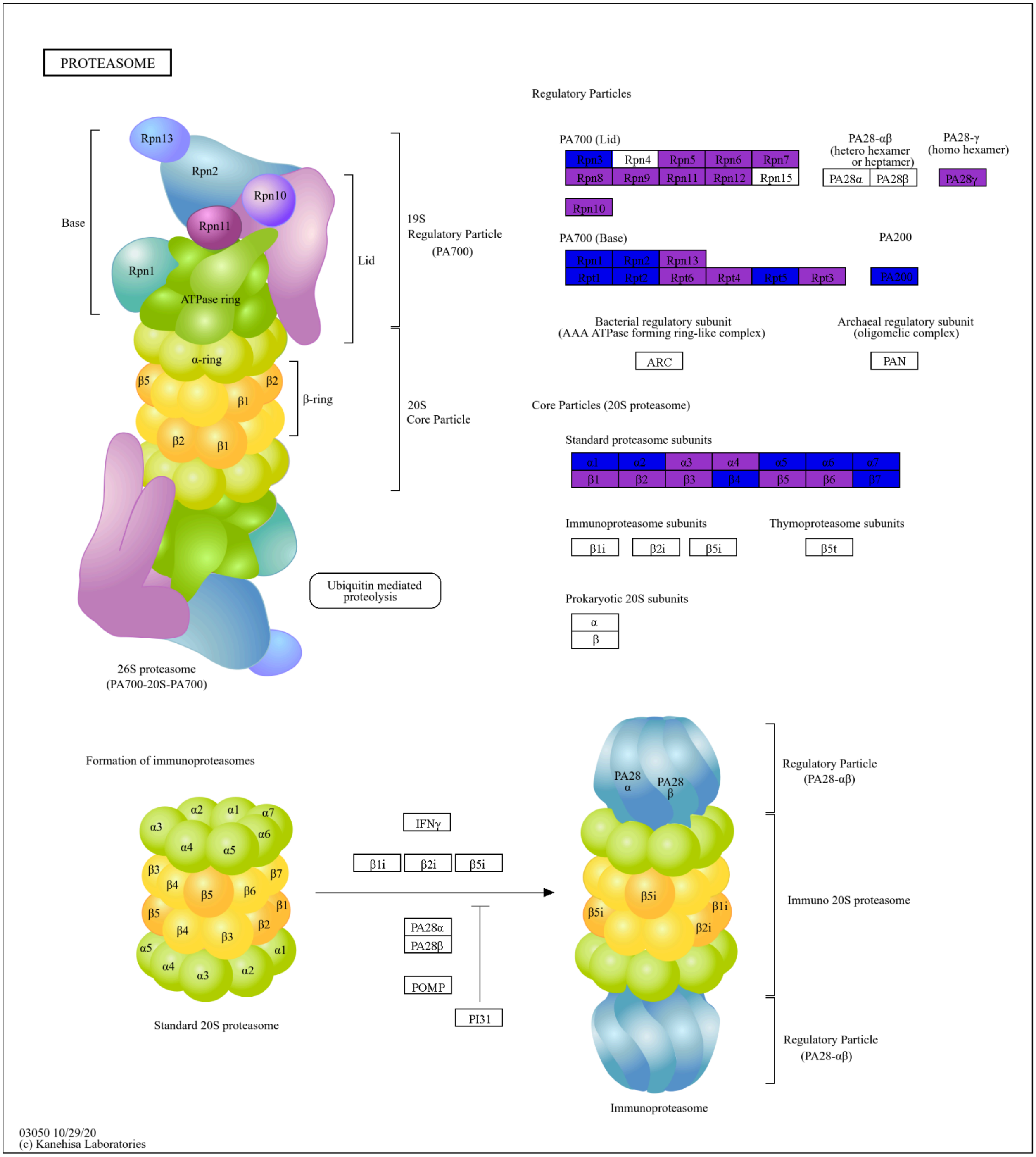

S8C Figure

**S8C Figure. KEGG map01212, fatty acid metabolism.** Proteins detected indicated as purple, proteins significantly downregulated at any timepoint indicated as blue, proteins significantly upregulated at any timepoint indicated as red.

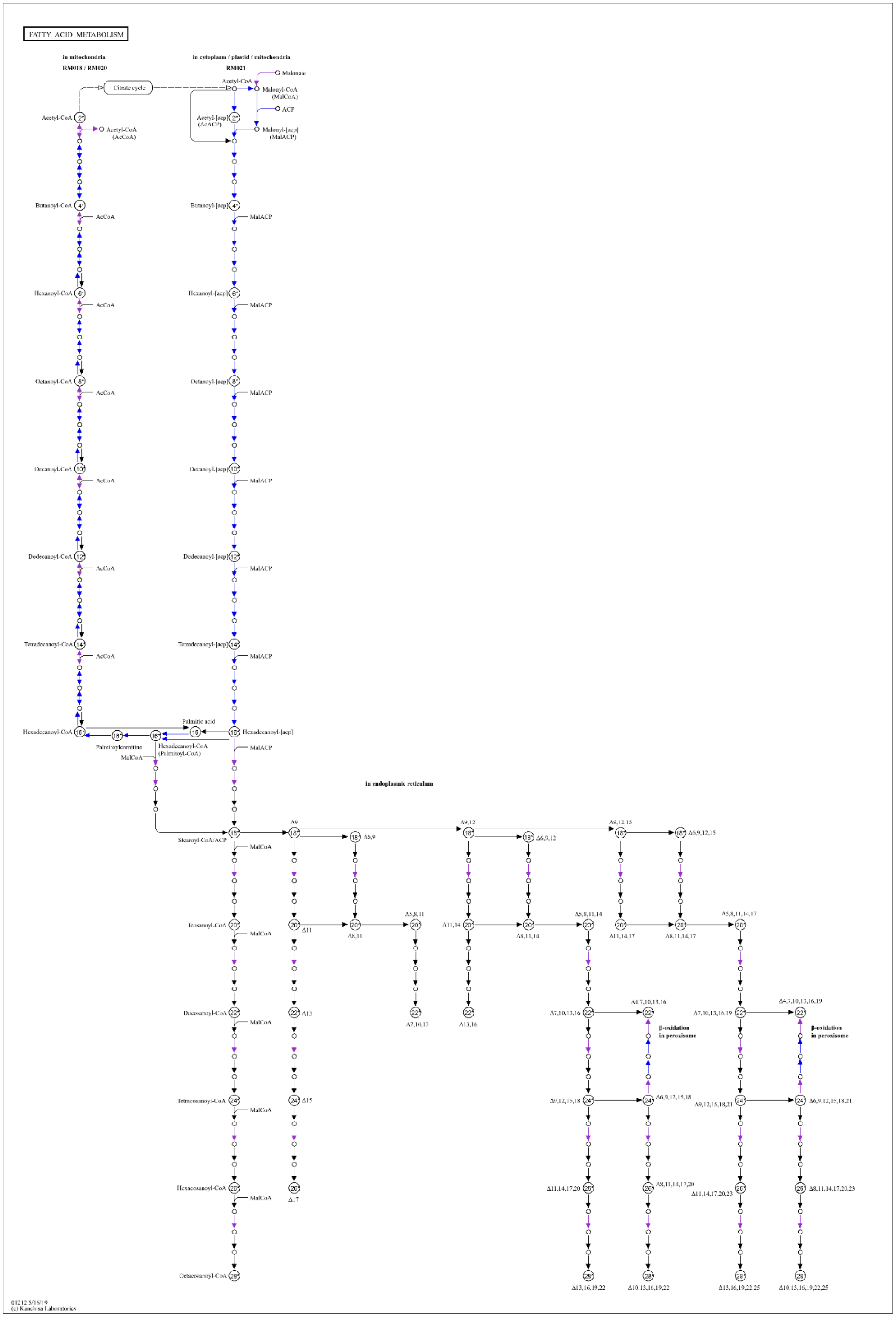

S8D Figure

**S8D Figure. KEGG map00900, terpenoid backbone biosynthesis.** Proteins detected indicated as purple, proteins significantly downregulated at any timepoint indicated as blue, proteins significantly upregulated at any timepoint indicated as red.

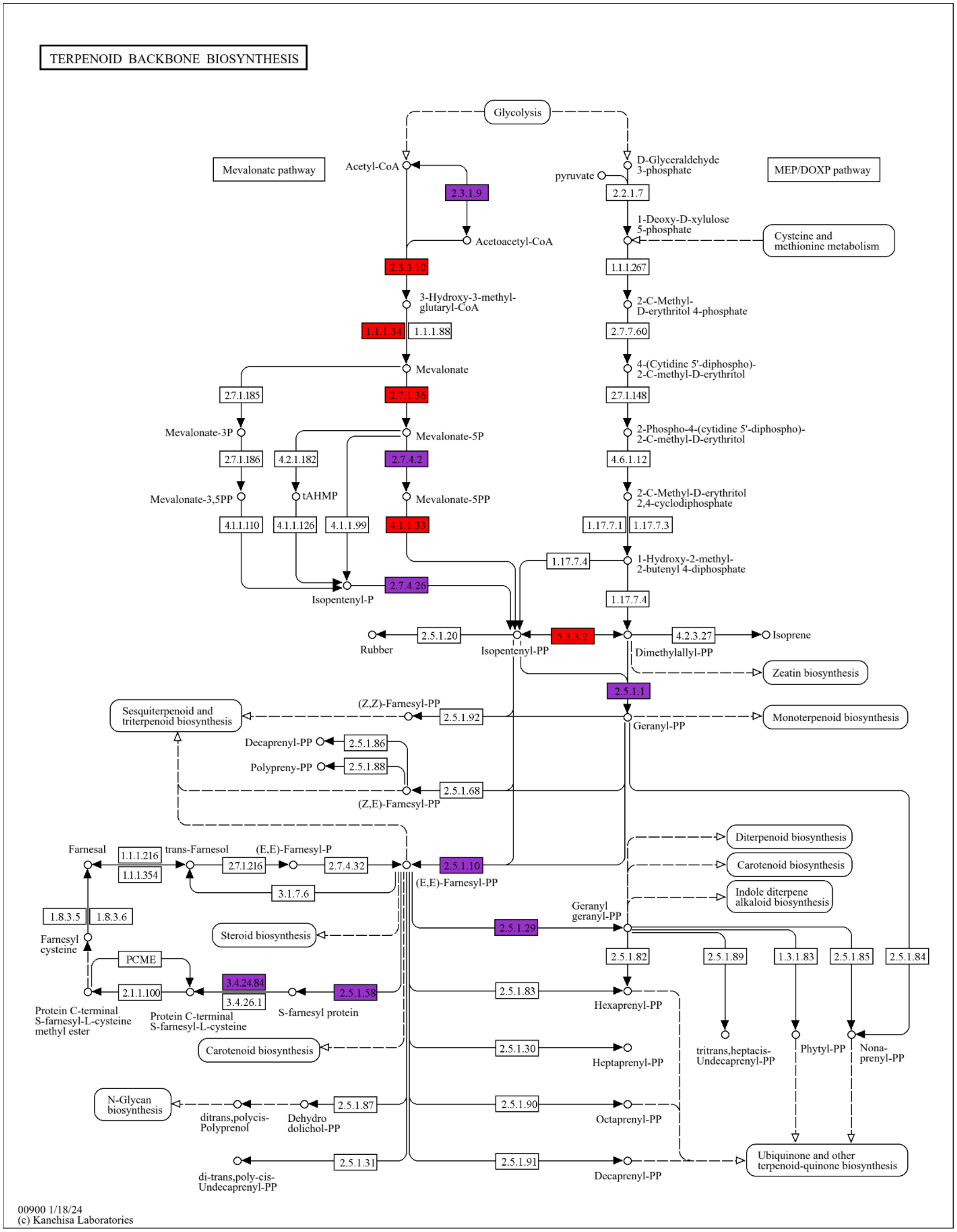

S8E Figure

**S8E Figure. KEGG map000010, Glycolysis / Gluconeogenesis.** Proteins detected indicated as purple, proteins significantly downregulated at any timepoint indicated as blue, proteins significantly upregulated at any timepoint indicated as red.

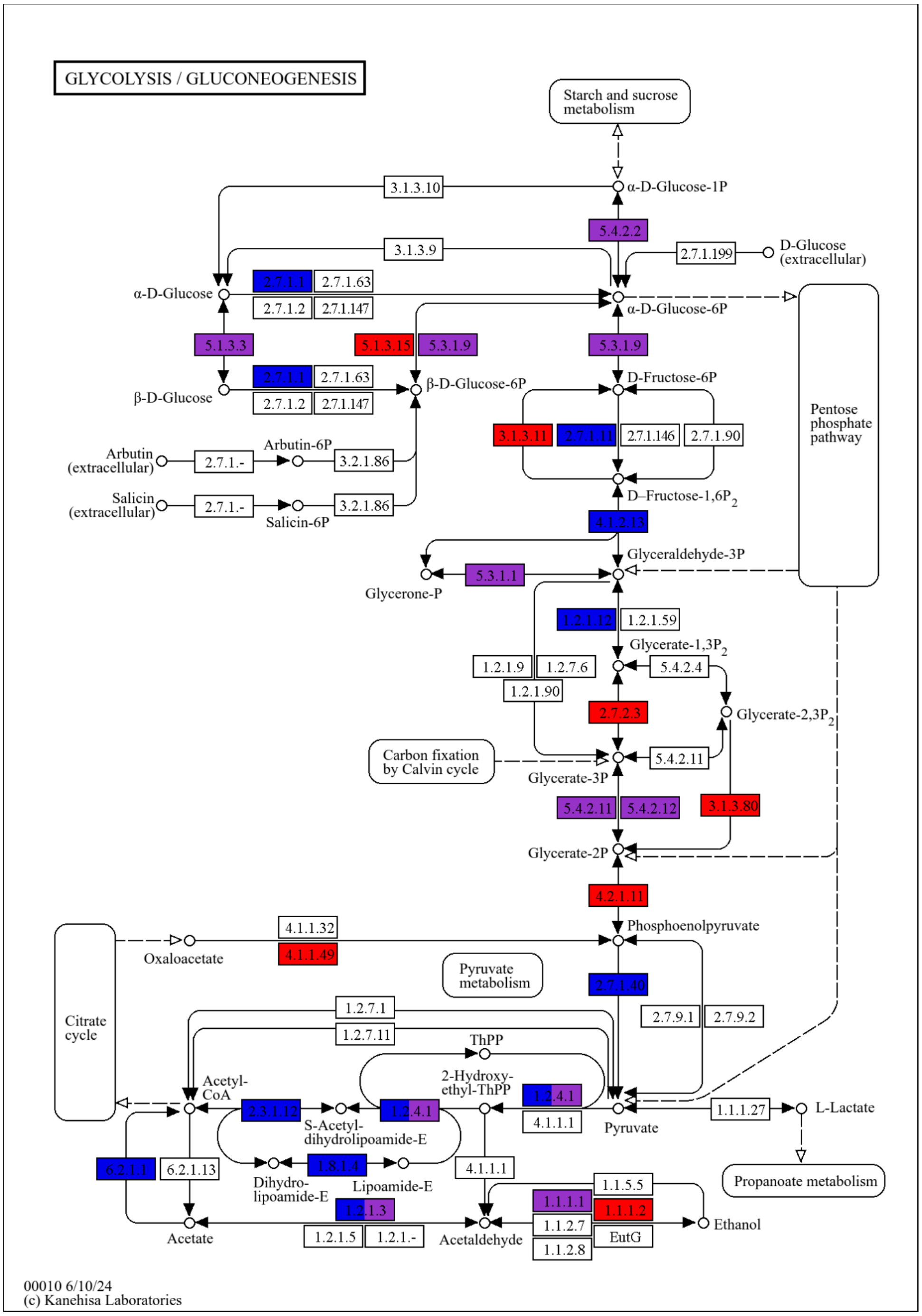

## S8F Figure

**S8F Figure. KEGG map000020, citrate cycle (TCA cycle).** Proteins detected indicated as purple, proteins significantly downregulated at any timepoint indicated as blue, proteins significantly upregulated at any timepoint indicated as red.

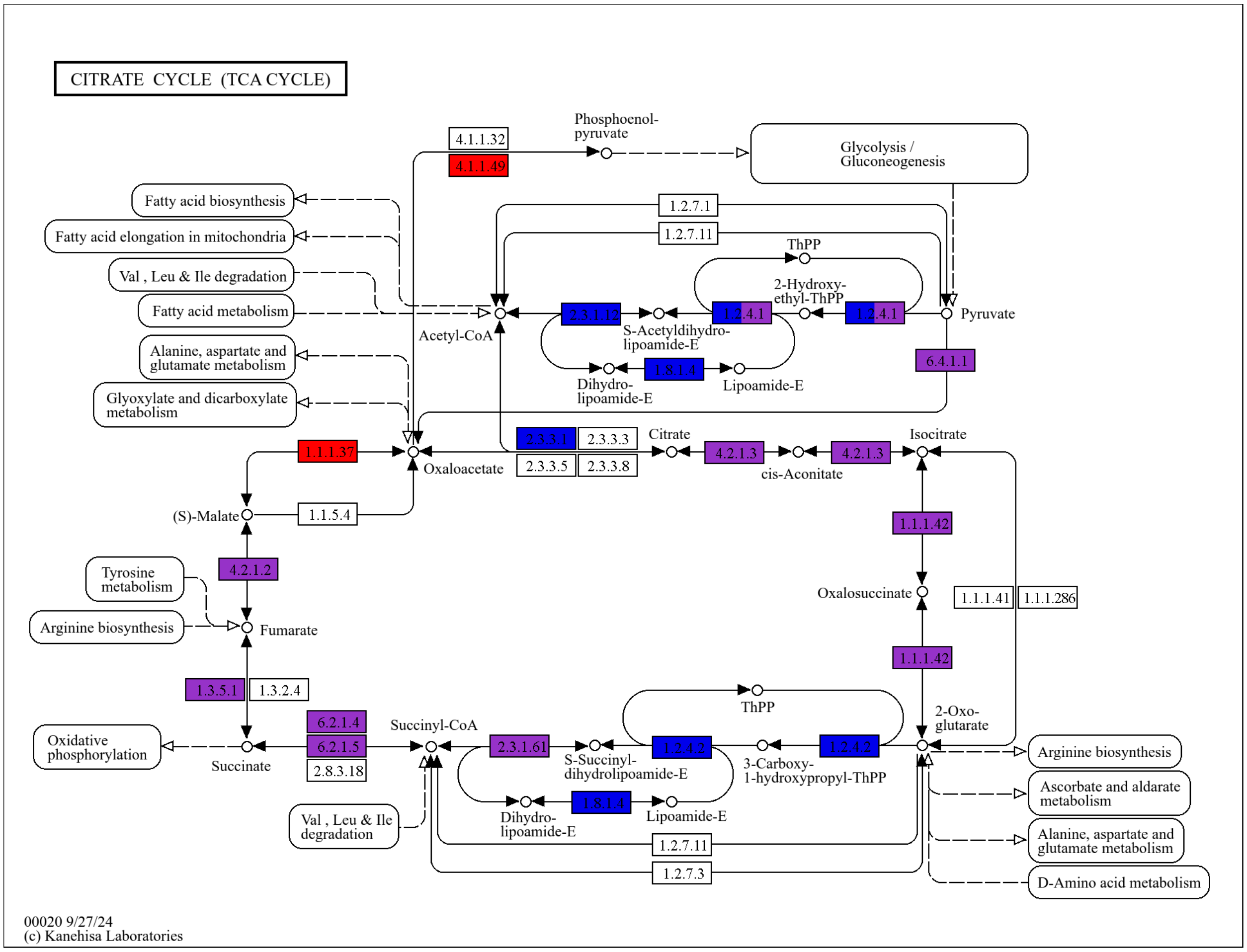

S8G Figure

**S8G Figure. KEGG map01230, amino acid biosynthesis.** Proteins detected indicated as purple, proteins significantly downregulated at any timepoint indicated as blue, proteins significantly upregulated at any timepoint indicated as red.

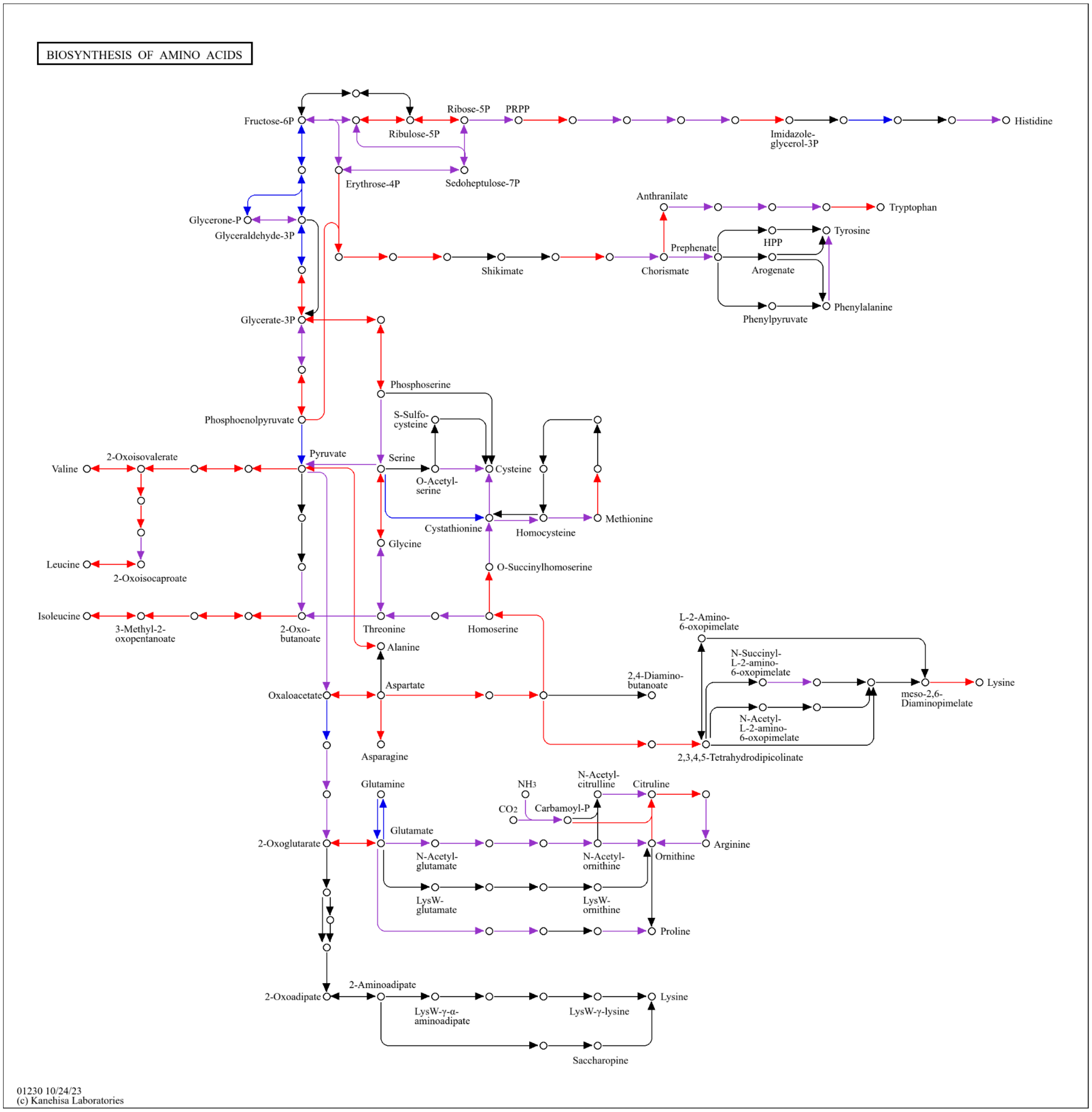

S8H Figure

**S8H Figure. KEGG map01232, nucleotide metabolism.** Proteins detected indicated as purple, proteins significantly downregulated at any timepoint indicated as blue, proteins significantly upregulated at any timepoint indicated as red.

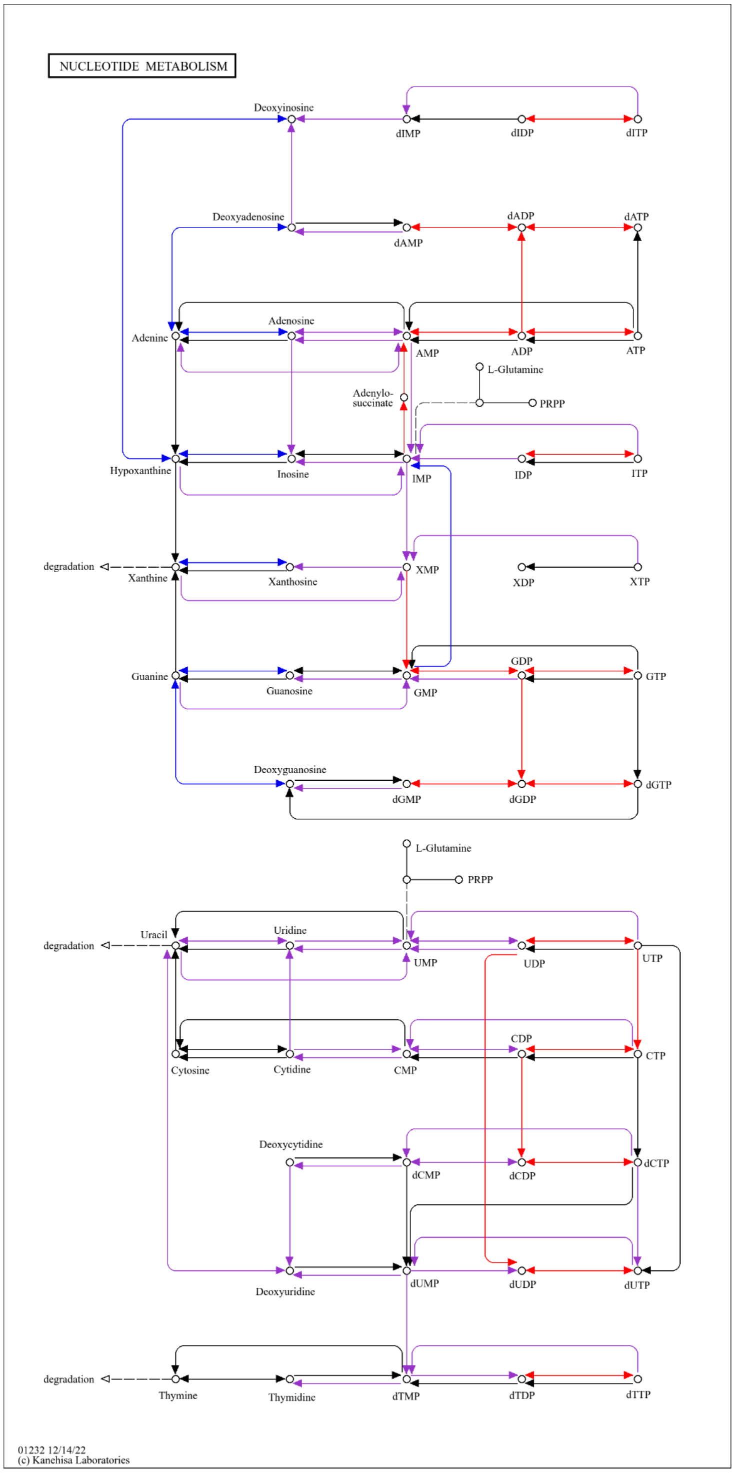

S8I Figure

**S8I Figure. KEGG map00670, One carbon pool by folate.** Proteins detected indicated as purple, proteins significantly downregulated at any timepoint indicated as blue, proteins significantly upregulated at any timepoint indicated as red.

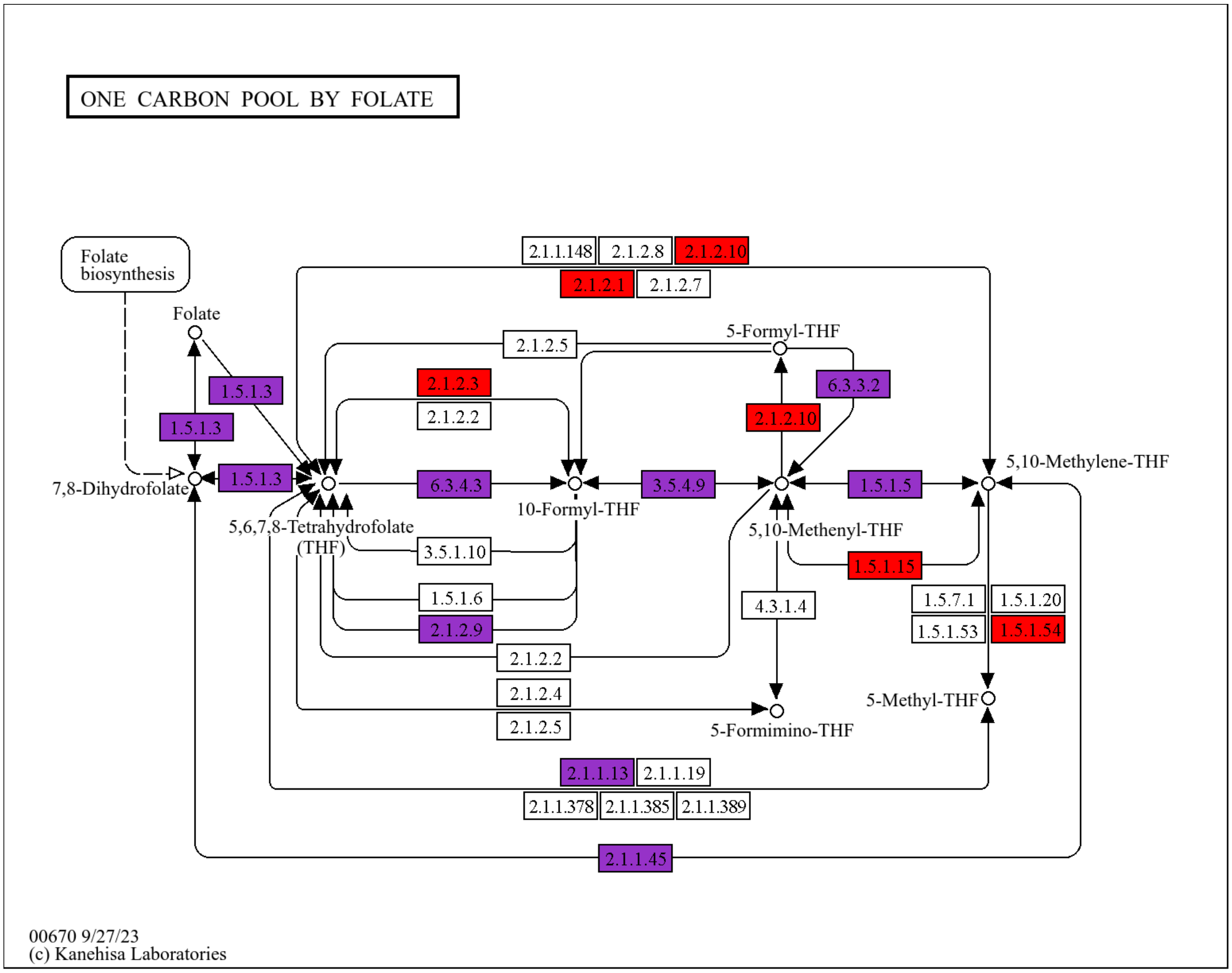

# S8J Figure

**S8J Figure. KEGG map00190, oxidative phosphorylation.** Proteins detected indicated as purple, proteins significantly downregulated at any timepoint indicated as blue, proteins significantly upregulated at any timepoint indicated as red.

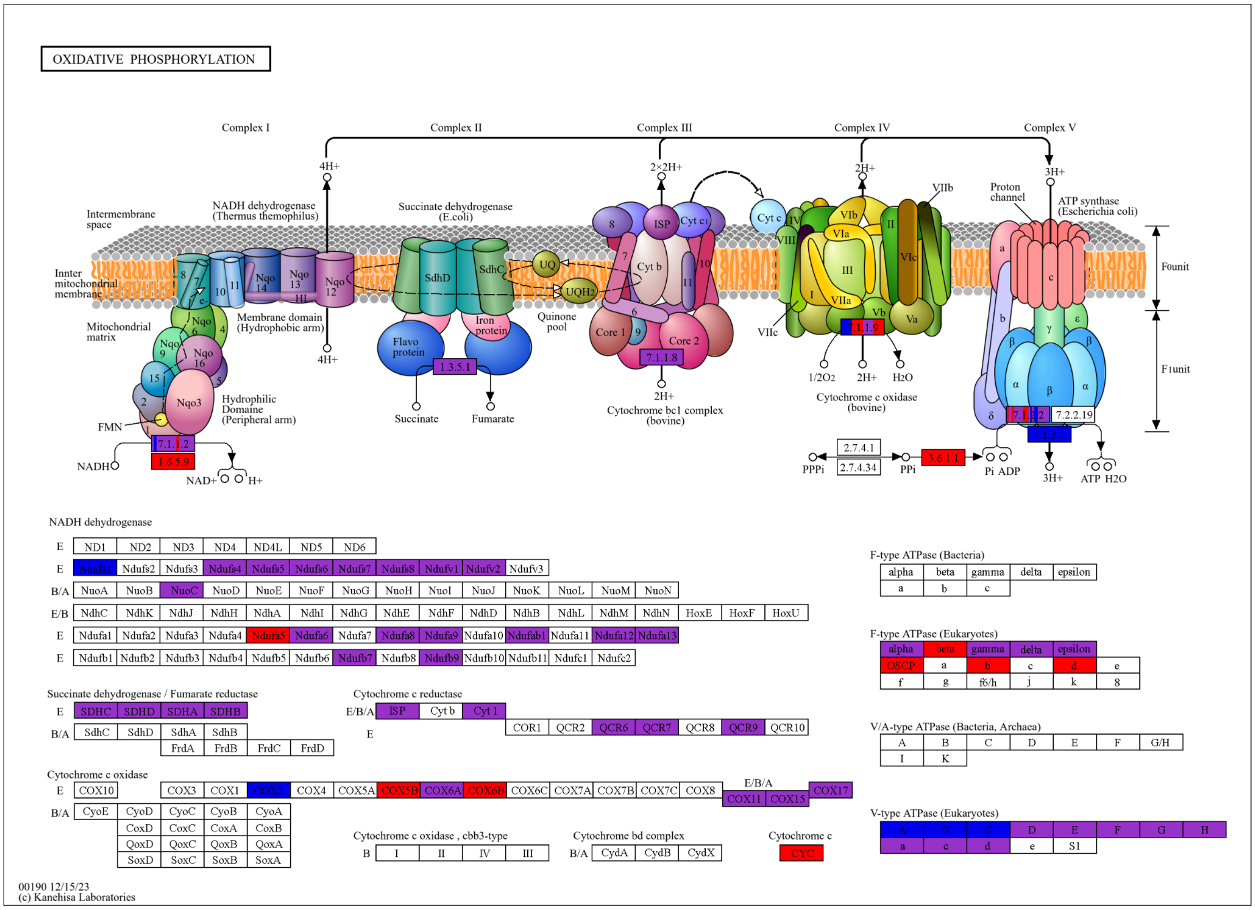

Supplement: S1 Appendix — (PDF) [file pone.0326651.s004.pdf]
